# Colloidal self-assembly based ultrathin metasurface for perfect absorption across the entire visible spectrum

**DOI:** 10.1515/nanoph-2022-0686

**Published:** 2023-01-12

**Authors:** Jiayi Jiang, Yan Cao, Xin Zhou, Haixia Xu, Kexin Ning, Xuan Xiao, Yanxin Lu, Cairong Ding, Yihang Chen, Jianwen Dong

**Affiliations:** Guangdong Provincial Key Laboratory of Quantum Engineering and Quantum Materials, School of Physics and Telecommunication Engineering, South China Normal University, Guangzhou 510006, China; State Key Laboratory of Optoelectronic Materials and Technologies, School of Physics, Sun Yat-sen University, Guangzhou 510275, China; School of Information Science and Technology, Zhongkai University of Agriculture and Engineering, Guangzhou 510225, China

**Keywords:** colloidal self-assembly, metasurface, perfect absorber, plasmonics

## Abstract

Perfect absorption over the entire visible spectrum can create a dark background for acquiring images with high contrast and improved resolution, which is crucial for various applications such as medical imaging, biological detection, and industrial non-destructive testing. The broadband absorption is desired to be achieved in an ultrathin structure for low noise as well as high integration. Here, we experimentally demonstrate a metasurface broadband perfect absorber with an ultrathin thickness of 148 nm and a large area of ∼10 cm^2^. Such a metasurface, with more than 97% absorption in the wavelength range from 400 to 800 nm, is composed of chromium nanodisk hexagonal array deposited on a chromium substrate with a silica spacer. A self-assembly based colloidal lithography nanofabrication method is developed for the scalable fabrication of the proposed nanostructure. We attribute the broadband absorption to the spectrally overlapped Fabry–Perot resonance, surface plasmon polariton, and localized surface plasmon resonances. Our results offer a novel approach to wafer-scale and low-cost manufacturing of absorption-based devices for applications such as high-contrast imaging and optical modulation.

## 1 Introduction

Dark-field photography is an important approach to acquire high contrast images for showing off the smooth curves of translucent or transparent objects. As an efficient imaging technique, it has been widely employed in medical and biological imaging, industrial non-destructive testing and security screening [1–3]. Through building a solid black background, dark-field photography can make observers see surface textures that otherwise be hidden using standard bright field. A direct and simple way to realize the dark background is using a broadband perfect absorber to eliminate the undesirable reflected or scattered light. The key criteria to assess the performance of such an absorber include the absorption strength in the visible region, as well as the volume of absorbing material. It is desirable to simultaneously possess high absorption intensity in the entire visible spectrum for generating high-contrast images, and thin material thickness for reducing noise [4, 5].

Metasurface is emerging as a promising candidate for broadband light absorber due to its unique and tailorable electromagnetic properties. Metasurfaces with nanoforest structures, such as carbon nanotubes or metal nanowires, can achieve broadband strong absorption by means of multilevel hybridized plasmon resonances and cavity mode resonances inside the hybrid nanoforest [6–9]. However, their large thickness (tens to hundreds of micrometres) impedes device integration and will cause relatively high intrinsic noise. Thin metasurface absorbers, which adopt metal-insulator-metal (MIM) configuration with subwavelength resonators, were recently extended to be broadband by using sophisticated schemes for blending resonant frequencies of individual resonating elements [10–17]. These complex nanostructures were fabricated predominantly by top-down lithography such as focused ion beam (FIB) milling and electron beam lithography (EBL), with inherent limitations on production throughput, available area, fabrication cost, and scalability [18]. Self-assembly based nanofabrication can provide a low-cost way to realize wafer-scale metasurfaces. Randomly dispersed [19, 20] or hexagonal densely arranged [21, 22] nanoparticles, combined with bottom layered substrate or upper deposited film, can lead to broadband perfect absorption [23, 24]. However, their structure thickness is larger than the diameter of the assembled nanoparticles, which makes it difficult to achieve ultrathin broadband absorbers.

Here, we demonstrate a broadband, polarization-independent ultrathin metasurface absorber with high feasibility for practical applications. Through the combined effect of Fabry–Perot resonance and surface plasmon resonances, the 148 nm-thick absorber, consisting of a chromium (Cr) nanodisk array, a silica (SiO_2_) spacer, and a Cr substrate, exhibits nearly perfect absorption in the entire visible spectrum. Our ultrathin device is promising for integrated, large-scale applications that require strong absorption in the visible region such as high-contrast imaging and optical modulation. In addition, we develop a self-assembled based nanofabrication method, using self-assembled colloidal nanoparticles as a mask followed by oxygen plasma etching and magnetron sputtering, to experimentally realize large-area (centimeter-scale) samples of the proposed broadband absorbers. Without any high-cost lithography (such as FIB, EBL, etc.), our cost-effective and scalable fabrication method provides an efficient way to high-throughput manufacturing of other nanophotonic structures and devices.

## 2 Design and fabrication

### 2.1 Structure design

**Figure 1:**
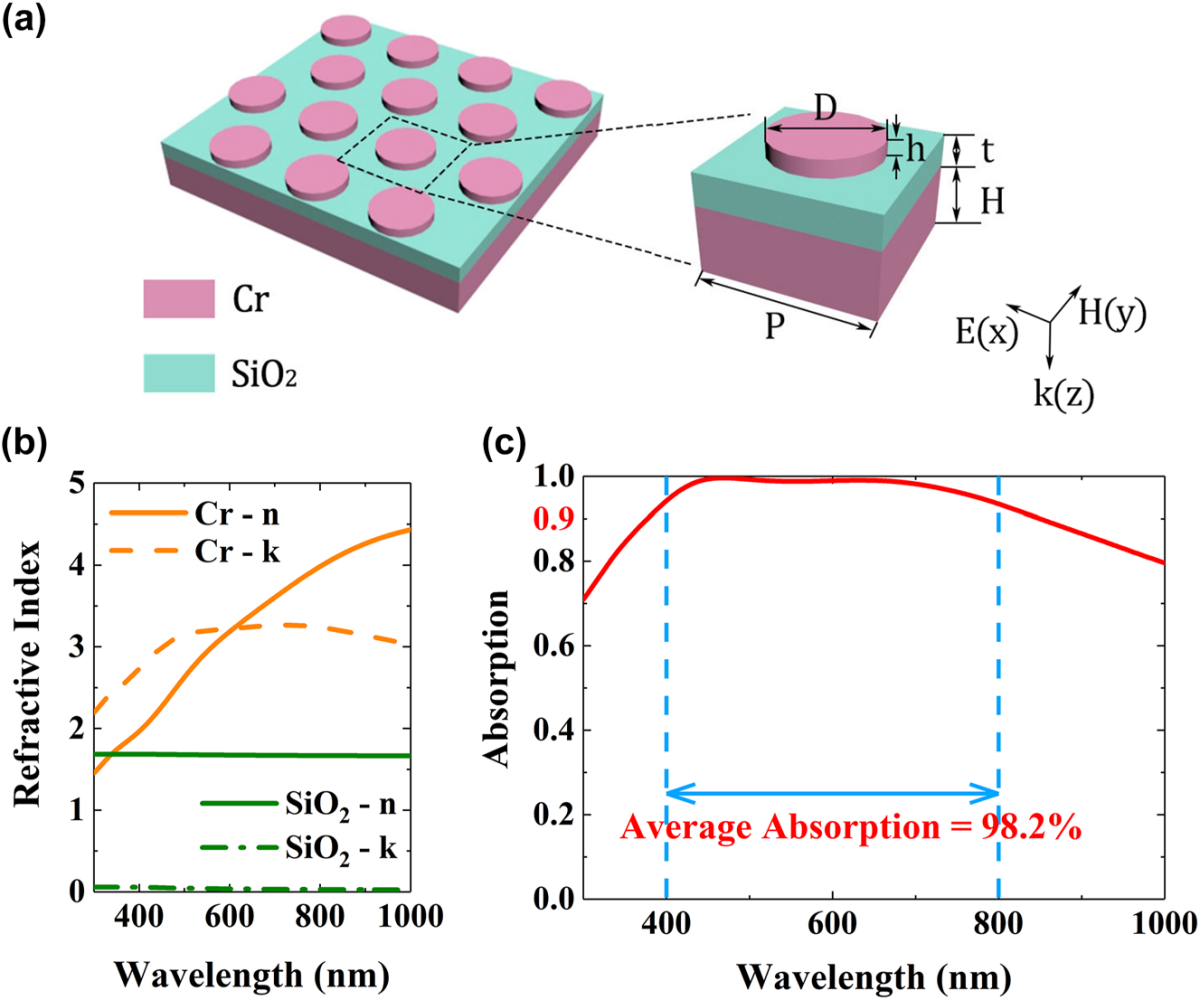
Design of broadband metasurface absorber. (a) Schematic of the broadband metasurface absorber. (b) Real and imaginary parts of the refractive indices of Cr and SiO_2_ measured from spectroscopic ellipsometry. (c) Simulated absorption spectrum of the metasurface absorber with *P* = 300 nm, *D* = 160 nm, *h* = 20 nm, *t* = 58 nm, and *H* = 70 nm.

The schematic of the proposed metasurface absorber is shown in Figure 1(a). Such a structure can be seen as a MIM stacked system. The top layer is composed of periodic chromium (Cr) nanodisk array with diameter *D*, height *h*, and period *P*, which is the core layer of the absorber, so that all incident electromagnetic wave energy enters the structure. The middle layer is a SiO_2_ dielectric spacer layer with the thicknesses *t*, where the incident electromagnetic wave is consumed. The bottom layer is metal chromium film with a thickness *H*. The optical constants of Cr and SiO_2_ are measured by the Cr and SiO_2_ films prepared by magnetron sputtering. Figure 1(b) shows the measured refractive indices of Cr and SiO_2_ using an ellipsometer. Solid and dashed lines are, respectively, the refractive index *n* and the extinction coefficient *k* at different wavelengths.

We perform the finite-difference time-domain (FDTD) simulation using the measured refractive indices to investigate the performance of the metasurface absorber with *P* = 300 nm, *D* = 160 nm, *h* = 20 nm, *t* = 58 nm, and *H* = 70 nm. The absorption is calculated by *A*(*λ*) = 1 − *R*(*λ*) − *T*(*λ*), where *R*(*λ*) denotes reflectance and *R*(*λ*) denotes transmittance. Assume that the thickness of the bottom Cr layer is much larger than the penetration depth so that the incident light is blocked and the transmission is zero (*T*(*λ*) = 0). The absorption spectrum of the metasurface absorber is shown in Figure 1(c). It is seen that a near-perfect absorption band covers the entire visible light region and it has an average absorption rate 98.2% from 400 to 800 nm.

### 2.2 Experimental section and results

**Figure 2:**
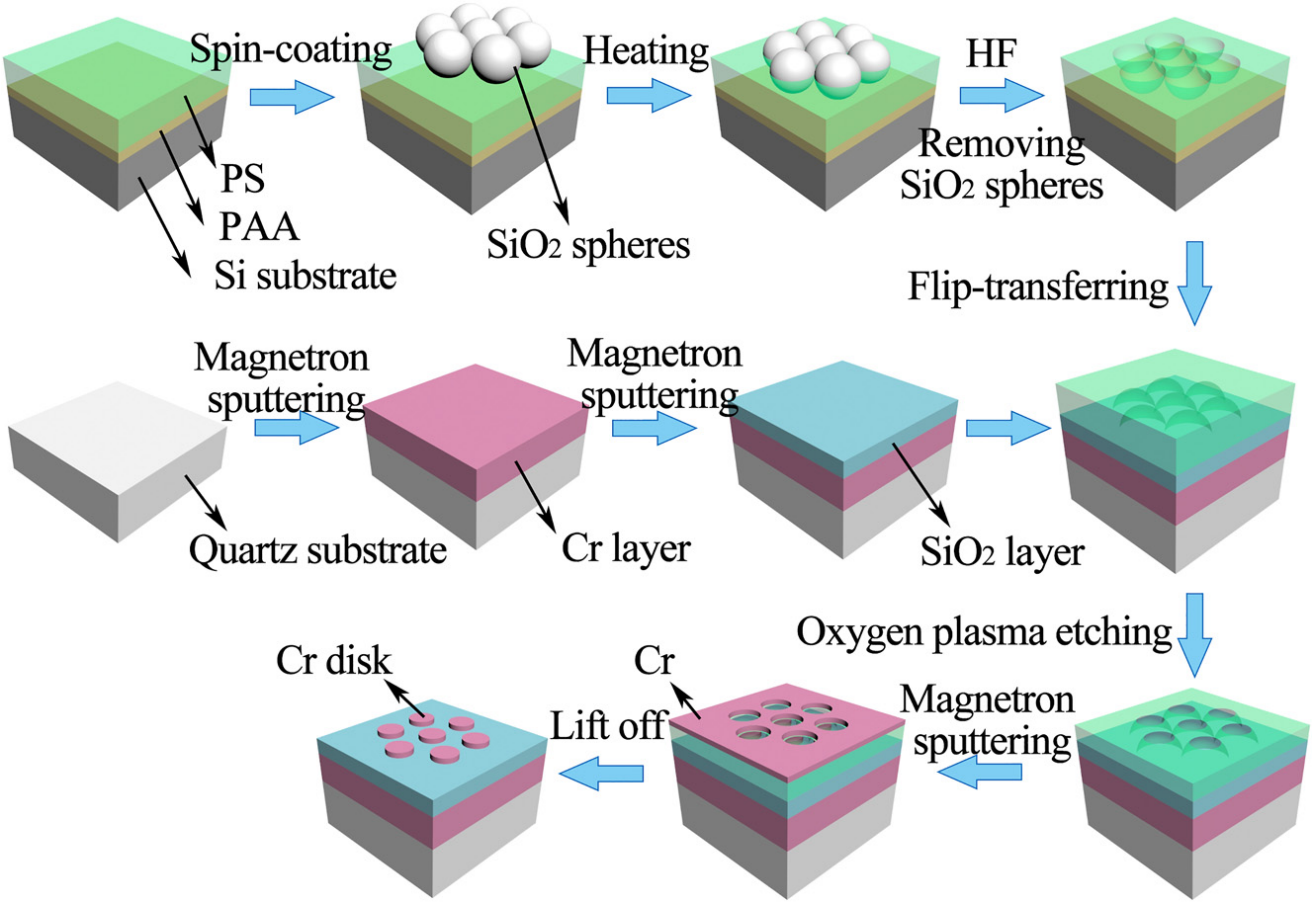
Scalable self-assembled-based fabrication scheme of the proposed metasurface absorber.

The proposed metasurface absorber is fabricated via a self-assembly based colloidal lithography method [25]. The fabrication scheme is shown in Figure 2.

First, a cleaned quartz substrate is deposited, in turn, with a 120 nm-thick Cr film and a 58 nm-thick SiO_2_ film through magnetron sputtering. When the vacuum of the chamber is lower than 1.3 × 10^−5^ Pa, argon (Ar, pure 99.999%) is pumped into the chamber and works as glow gas. The Cr film is deposited with RF power of 50 W and pressure of 2.5 × 10^−2^ Pa, and the SiO_2_ film is deposited with RF power of 120 W and pressure of 2.2 × 10^−2^ Pa. The Cr/SiO_2_ structure is regarded as the substrate of the visible light metasurface absorber.

**Figure 3:**
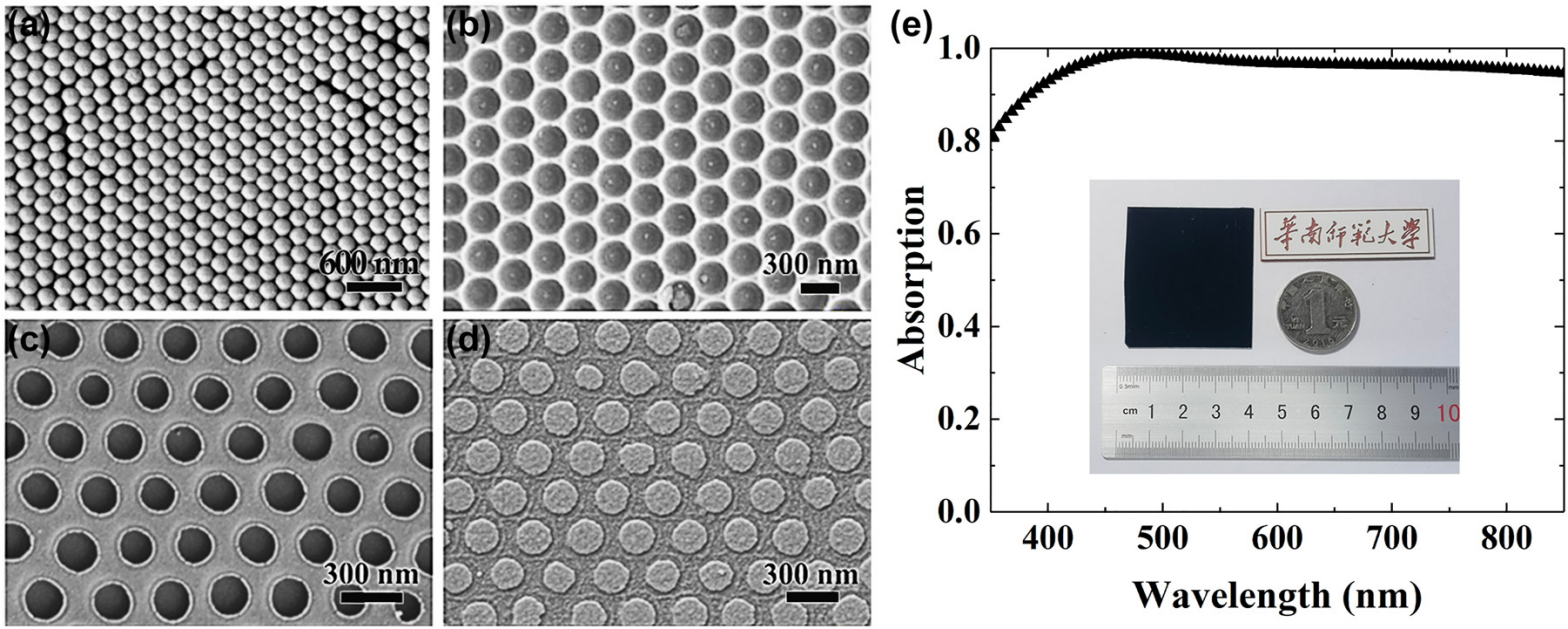
Structural characterization and measured spectrum. Scanning electron microscope (SEM) images of (a) hexagonal close-packed SiO_2_ nanosphere array, (b) hemispherical dimple honeycomb array in the PS film, (c) nanopore array in the PS film, and (d) periodic Cr nanodisk array. (e) Measured absorption spectrum of the absorber sample. The inset shows the camera image of the sample.

Second, the mask was fabricated as follows: a cleaned silicon substrate is spin coated, in turn, with a layer of sacrificial polyacrylic acid (PAA, 3 wt%) film at 1000 rpm for 37 s and the toluene solution of polystyrene (PS, 1.7 wt%) film at 2000 rpm for 37 s. Next, the hydrophilicity of the PS surface modification is made by oxygen plasma etching at the oxygen flow 6 sccm and the power 80 w for 40 s. Then, SiO_2_ solution with a diameter of 300 nm and a concentration of 20% is spin coated on the PS film at 300 rpm for 5 s and at 600 rpm for 35 s. A large-area, short-range orderly hexagonal close-packed SiO_2_ microsphere array is formed, as shown in Figure 3(a). Next, we place the sample on a heating stage at 120 °C for 4.5 min, so that the SiO_2_ microspheres are embedded into the PS film. Then, the sample is immersed in diluted hydrofluoric acid (HF) solution for 20 min in order to remove the SiO_2_ microsphere, forming a periodically arranged honeycomb array, as shown in Figure 3(b). The depth of the SiO_2_ microspheres embedded into the PS film depends on the heating time. However, the heating time should not be too long so that the SiO_2_ microspheres will not penetrate the PS film. Then, the sample is immersed in distilled water, and the PAA film is dissolved to separate the PS film from the silicon wafer. The PS film is floating on the water.

Third, the PS film is flip-transferred to the pre-fabricated Cr/SiO_2_ substrate of the metasurface absorber. Then, the PS film is gradually etched in a downward direction by oxygen plasma etching to obtain a periodic nanopore array, as shown in Figure 3(c). The diameter of the nanopore is determined by the etching time.

Finally, a Cr film of 20 nm-thick is deposited on the nanopore template by magnetron sputtering. After the lift-off process to remove the PS template with chlorobenzene, the periodic array of Cr nanodisk is fabricated, as shown in Figure 3(d). It is seen that the Cr nanodisk array has good symmetry, the ring traces on the surface are a small amount of residual PS film.

To characterize the fabricated metasurface absorbers, we use Maya 2000 Pro VIS-NIR spectrometer to measure the absorption of the samples. Figure 3(e) shows the measured absorption spectrum of the absorber sample with structural parameters the same as those in Figure 1. It is seen that the sample exhibits very strong absorption across the whole visible region, achieving an average absorption of 97.1% in a wide wavelength range from 400 to 800 nm. Overall, the measured result agrees well with the simulations. The slight difference between the simulated and the measured absorption spectra is mainly resulted from the deviation of the size of the fabricated Cr nanodisks from that of the designed ones. The inset in Figure 3(e) shows the image of an absorber sample taken under indoor ambient light illumination. It can be seen that the nearly perfect broadband absorption is achieved in the sample of about 10 cm^2^ area.

**Figure 4:**
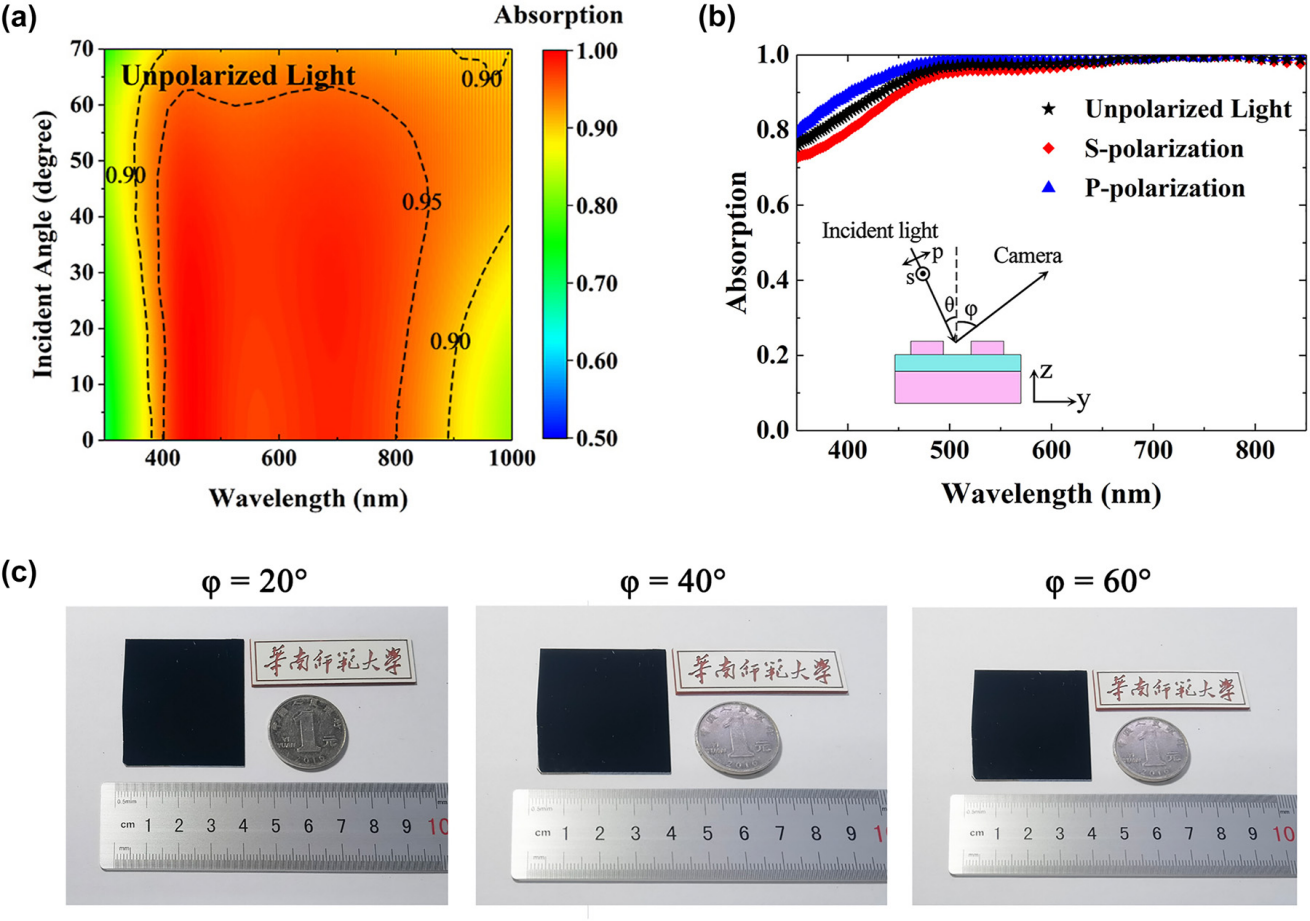
Omnidirectional absorption characterization. (a) Absorption spectrum versus incident angle for the metasurface absorber under unpolarized illumination. (b) Absorption spectra of the metasurface absorber at incident angle of 30° for s-polarized, p-polarized, and unpolarized light. (c) Digital camera images of the absorber sample taken at angles of 20°, 40°, 60°.

We further investigate the influence of the incident angle and polarization on the absorption performance of our device, as shown in Figure 4. It is seen from Figure 4(a) that the performance of our metasurface absorber exhibits a good angular tolerance up to 70°. The average absorption across the entire wavelength range of 400–800 nm is 95.4% for our absorber illuminated at angle of 60°. Polarization-insensitive absorption performance of our device can be observed in Figure 4(b) where the absorption spectra at incident angle of 30° under s-polarized, p-polarized, and unpolarized illumination are presented. Figure 4(c) presents the digital camera images of the absorber sample taken at different angles**.** The sample appears solid black even at large viewing angle of 60°.

Note that such a strong absorption occurs in a 148 nm-thick structure. Average absorption in the entire visible region and structure thickness are two key performance metrics for absorbers applied to high-contrast and low-noise imaging. In Table 1, we list the key performance indicators of the state-of-the-art broadband absorbers working at visible wavelengths. It is seen that our device attains a record-high value of average-absorption/structure-thickness ratio among these broadband absorbers in the visible spectrum.

**Table 1:** Comparison of the state-of-the-art broadband absorbers working in the visible region.

	Average absorption	Structure	Average absorption/	Fabrication methods	Scalable
	in the entire visible	thickness	structure		manufacture
	spectrum	(nm)	thickness (nm^−1^)		
Li [5]	0.91	∼2000	<4.6 × 10^−4^	Cu^2+^ ion etching, two-step Boehmite treatment, dip-coating	Yes
Kiani [6]	0.99	∼30,000	<3.3 × 10^−5^	Thermal oxidation growth, hydrogen thermal reduction, self-catalytic chemical vapor deposition	Yes
Kim [8]	0.91	40,040	2.3 × 10^−5^	Capillary forced lithography, high step-coverage deposition, dry etching	Yes
Jia [10]	0.96	240	4.0 × 10^−3^	Magnetron sputtering, electron beam evaporation, electron beam lithography, dry etching	No
Chaudhuri [14]	0.90	490	1.8 × 10^−3^	Electron beam evaporation, electron beam lithography, dry etching	No
Qin [16]	0.94	650	1.4 × 10^−3^	Magnetron sputtering, ion beam sputtering, lithography, electron beam evaporation	No
Zhou [18]	0.99	1085	9.1 × 10^−4^	Two-step anodization method, template-assisted physical vapor deposition	Yes
Lee [21]	0.95	268	3.5 × 10^−3^	Colloidal self-assembly, reactive ion etching, electron beam evaporation	Yes
Hou [23]	0.98	685	1.4 × 10^−3^	Colloidal self-assembly, reactive ion etching, magnetron sputtering	Yes
Søndergaard [26]	0.96	500	1.9 × 10^−3^	Magnetron sputtering, focused ion beam	No
Lin [27]	0.95	1030	9.2 × 10^−4^	Laser writing, self-assembly graphene oxide coating, photo-induced reduction	No
Our work	0.97	148	6.6 × 10^−3^	Colloidal self-assembly, oxygen plasma etching, magnetron sputtering	Yes

## 3 Discussion on absorption mechanism

**Figure 5:**
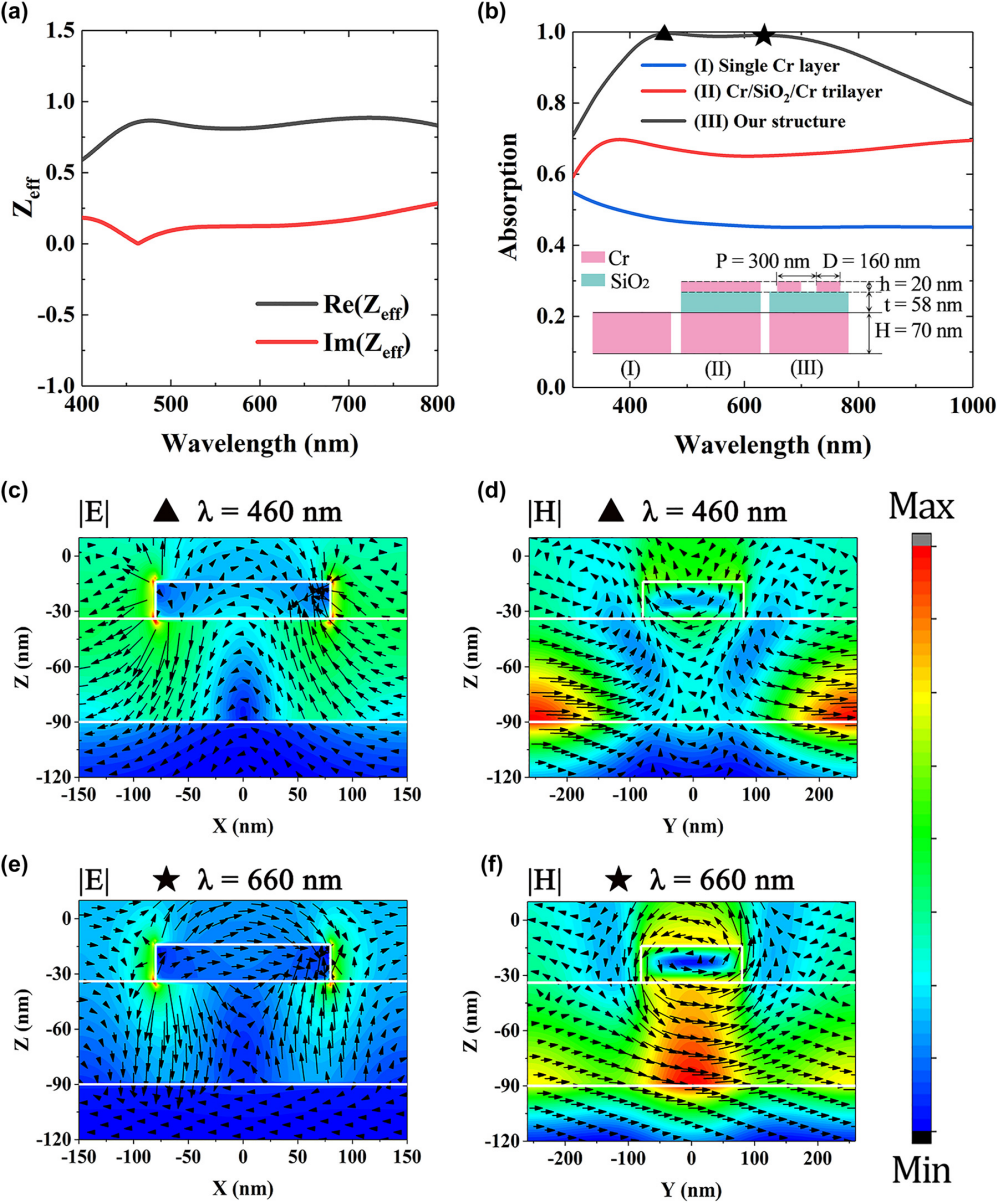
Illustration of absorption mechanism. (a) Impedance of the metasurface absorber. (b) Simulated absorption curves of bare Cr, Cr/SiO_2_/Cr trilayer, and our absorber. (c)–(f) Distributions of the electric field (E) (color maps) and the electric vectors (arrow maps) of the structure in *x*–*z* plane at (a) 460 nm and (c) 660 nm. Distributions of the magnetic field (H) and the magnetic vectors of the structure in *y*–*z* plane at (b) 460 nm and (d) 660 nm.

We calculated the impedance of the considered metasurface using the S-parameter retrieval algorithm [28, 29]:

(1) $$z_{\text{eff}}=\sqrt{\frac{\mu_{\text{eff}}}{\varepsilon_{\text{eff}}}}=\pm \sqrt{\frac{{\left(1+S_{11}\right)}^{2}-{S_{21}}^{2}}{{\left(1-S_{11}\right)}^{2}-{S_{21}}^{2}}}$$

where *μ*_eff_ and *ɛ*_eff_ are the effective permeability and permittivity, respectively. The results are shown in Figure 5(a). It is seen that $Z_{\text{eff}}\left(Re\right)\approx 1$ and Z_eff_(Im) ≈ 0 in the wavelength range of 400–800 nm, i.e. the impedance of the metasurface can almost match to that of vacuum in the whole visible region, and consequently the wide absorption band forms.

To reveal the physical mechanism of the broadband absorption, we show the absorption spectra of a single Cr thick layer, a Cr/SiO_2_/Cr trilayer, and our metasurface in Figure 5(b). It is seen that wideband absorption exists for the Cr thick layer because of its relatively large extinction coefficient. However, the absorption does not exceed 0.5 in the visible region. For the Cr/SiO_2_/Cr trilayer, the top and bottom Cr layers can be regarded as two opposite mirrors and they construct a resonant cavity. When light is vertically incident, Fabry–Perot (FP) resonance can be excited in the trilayer structure, resulting in the enhancement of absorption as shown in Figure 5(b). When the top Cr layer is replaced by the Cr nanodisk array, the absorption further increase because of the surface plasmon resonances. It is also seen that the absorption of the metasurface absorber reaches maxima at around 460 and 660 nm, respectively.

We then simulated the electric and magnetic field distributions in our absorber at wavelengths of 460 and 660 nm, as shown in Figure 5(c)–(f). It is seen from Figure 5(c) and (d) that the electric field is mainly localized at near both ends of the Cr nanodisk, while the magnetic field is localized in the gaps between the nanodisks. These field patterns agree with those of propagating surface plasmon (PSP), indicating the absorption peak around 460 nm is mainly contributed by the PSP resonance. Figure 5(e) and (f) show the electric and magnetic field distributions at wavelength of 660 nm. It is observed that strong field localization exists near the lower-ends of the Cr nanodisk and the magnetic vectors form a loop around the Cr nanodisk, indicating that an electric dipole exists (see Supplementary data 1 for details), which is consistent with the field local characteristics of localized surface plasmon (LSP) resonance. In addition, anti-parallel current forms inside the SiO_2_ layer and the magnetic field is strongly confined in the region below the Cr nanodisk, which is the symbol of the magnetic polariton (MP) resonance [22, 30, 31]. In this case, our metasurface structure can be equivalent to an LC circuit model and each unit cell can be regarded as a magnetic dipole (see Figure S2). When the total impedance of the LC model is zero, MP resonance can significantly enhance the absorption.

**Figure 6:**
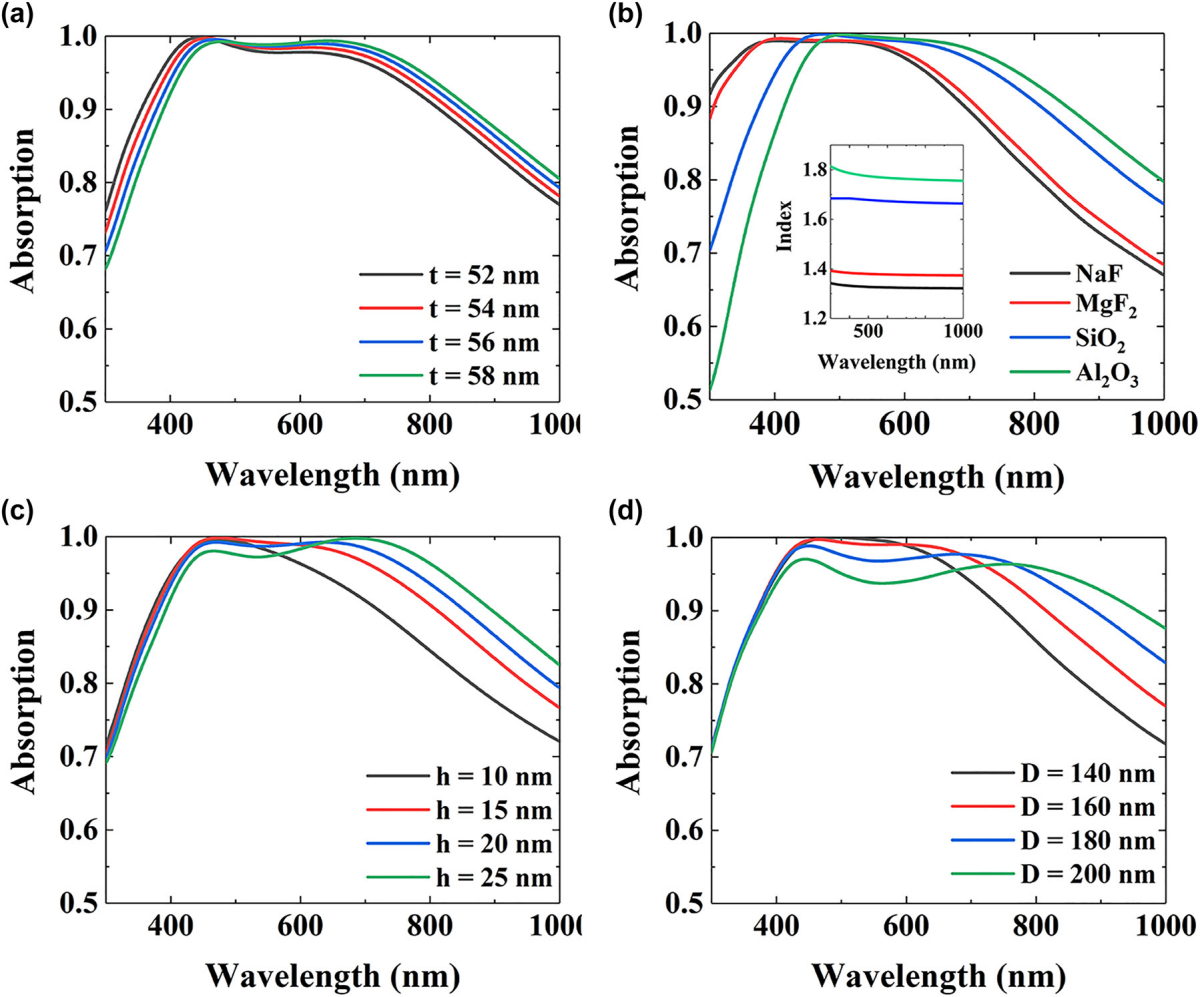
The absorption spectra of the metasurface absorber at different (a) *t*, (b) dielectric material, (c) *h*, (d) *D*. The other structural parameters are the same as those in Figure 1.

We also investigate the influence of the structural parameters on the performance of our metasurface absorber, as shown in Figure 6. Figure 6(a) and (b) show the simulated absorption spectra of the proposed metasurface absorber under different thickness *t* and materials of the dielectric spacer. As shown in Figure 6(a) and (b), with the increase of the thickness *t* or refractive index *n* of the dielectric spacer, a red shift of the absorption band can be observed. As the FP resonance exists between the top Cr nanodisks and the bottom Cr substrate, the increase of *t* or *n* leads to the increase of the optical path in the dielectric layer, thereby resulting in the red shift of the absorption band. Figure 6(c) and (d) show the absorption spectra of the proposed metasurface absorber under different height *h* and diameter *D* of the Cr nanodisks. It can be seen that as *h* or *D* increases, the position of the absorption peak at shorter wavelength remains almost unchanged, while the absorption peak at longer wavelength exhibits an obvious red shift. The reason for these phenomena is that the resonance wavelength of MP resonance increases with the increase of *h* or *D* (see Appendix A for details). Consequently, the broadband near-perfect absorption in our device is induced from FP, PSP, LSP and MP resonances.

## 4 Conclusions

In summary, we reported a large area, ultrathin broadband visible light metasurface absorber composed of periodic Cr nanodisk array, a SiO_2_ film spacer, and a Cr substrate. The broadband perfect absorption was attributed to the combined effect of the Fabry–Perot resonance, the propagating and localized surface plasmons. The 148 nm-thick metasurface absorber has an average absorption over 97% in the wavelength range from 400 to 800 nm, which makes it suitable for absorption-based applications such as high-contrast medical and biological imaging. Our metasurface absorber is fabricated by a cost-effective, scalable, high-throughput self-assembly based process that does not require any lithography. With the development in the design and fabrication of different masks and templates, we expect that the self-assembly based nanofabrication technique will open up new scalable processing schemes for the large-scale manufacture of complex nanostructures, which could lead toward a diverse set of other applications, such as plasmonic lasers [32], nonlinear nanophotonic devices [33, 34], nanodevices with ultra-high *Q* [35], compact high-resolution color displays [36], etc.

## Supplementary Material

Supplementary Material Details
