# Supplementary material for: Colloidal self-assembly based ultrathin metasurface for perfect absorption across the entire visible spectrum
Source: Nanophotonics. 2023 Jan 12;12(8):1581–90. doi: 10.1515/nanoph-2022-0686 (PMC11502047; doi:10.1515/nanoph-2022-0686)
Supplement: Supplementary file 1 — Supplementary Material Details [file j_nanoph-2022-0686_suppl.docx]

Jiayi Jiang, Yan Cao, Xin Zhou, Haixia Xu, Kexin Ning, Xuan Xiao, Yanxin Lu, Cairong Ding, Yihang Chen* and Jianwen Dong*

Colloidal self-assembly based plasmonic metasurface for perfect absorption across the entire visible spectrum

Supplementary data

1. Criteria for determining the LSP resonance

It is seen from Fig. S1 that an electric dipole forms at around the Cr nanodisk, which is consistent with the characteristic of LSP resonance.


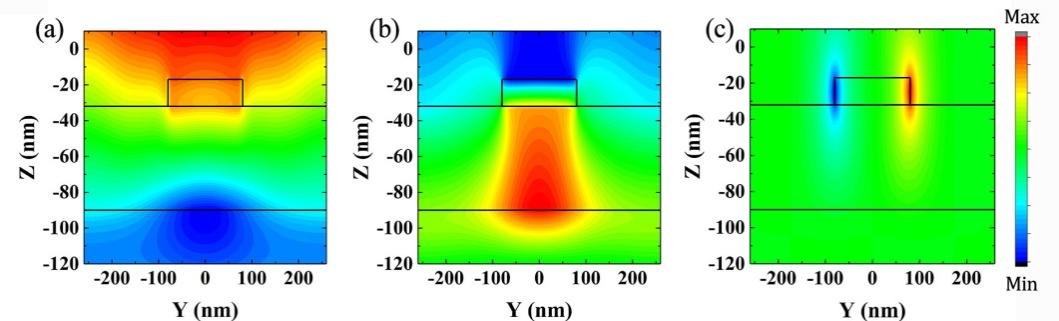


**Fig. S 1**: Distributions of (a) E_x_, (b) H_y_, (c)H_z_ in the y-z plane at 660 nm for the metasurface absorber shown in Fig. 1.

1. Discussions on the dependence of the MP resonance on the height h and diameter D of the Cr nanodisks

Fig. S2 shows the equivalent LC circuit for a unit cell of the proposed absorber structure. In Fig. S2, Lm is the inductance of the metal structures and it can be expressed as $L_{m}=0.5\mu_{0}t$, where $\mu_{0}$ is the permeability of vacuum and *t* is the thickness of the SiO_2_ spacer. C_m_ is the parallel-plate capacitance between the top and the bottom metal layers and it satisfies$C_{m}=\frac{c_{1}\varepsilon_{2}\varepsilon_{0}D^{2}}{t}$, where $\varepsilon_{2}$ and $\varepsilon_{0}$ are the permittivities of vacuum and SiO_2_, respectively. c_1_ is a semi-empirical constant which takes into account the fringe effect or non-uniform charge distribution along the surface of the capacitor. $L_{e}=\frac{1}{\varepsilon_{0}{\omega_{p}}^{2}}$ is the kinetic inductance that originates from the kinetic energy of free electrons in Cr, $\omega_{p}$is the plasma frequency of Cr, δ is the effective penetration depth and it is determined by $=\frac{1}{2\kappa\omega}$, where *κ* is the extinction coefficient and $\omega$ is the angular frequency of the incident light. At the resonant frequency of MP, can be written as ${}_{R}=\frac{1}{2\kappa\omega_{R}}$. $C_{g}=\frac{\varepsilon_{0}\mathrm{hD}}{P-D}$ represents the gap capacitance among the Cr nanodisks. The total impedance of such a LC circuit model is [1-3]

$Z_{\mathrm{tot}}=\frac{L_{m}+L_{e}}{1-{\omega_{R}}^{2}C_{g}(L_{m}+L_{e})}-\frac{2}{{\omega_{R}}^{2}C_{m}}+\left( L_{m}+L_{e} \right).$ (2)

$Z_{\mathrm{tot}}=0$ should be satisfied at the MP resonance frequency and thus, the resonance frequency can be written as

${\omega_{R}}^{2}=\frac{C_{g}+C_{m}\pm\sqrt{{C_{g}}^{2}+{C_{m}}^{2}}}{C_{m}C_{g}(L_{m}+L_{e})}.$ (3)


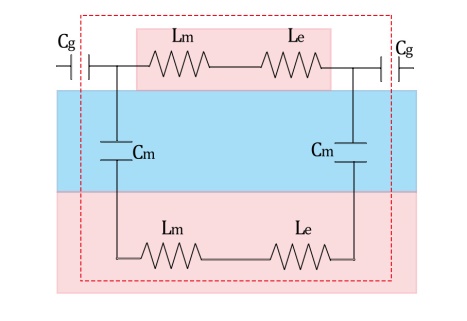


**Fig. S 2**: Schematic of the equivalent LC circuit for a unit cell of our metasurface absorber.

Here, we investigate the dependence of $\omega_{R}$ on the diameter (*D*) of the Cr nanodisks. Since $L_{m}$ is independent of *D*, we have

${\omega_{R}}^{2}\propto\frac{C_{g}+C_{m}\pm\sqrt{{C_{g}}^{2}+{C_{m}}^{2}}}{C_{m}C_{g}}\cdot\frac{1}{L_{e}}$. (4)

By substituting the expression of Le into Eq. (4), we have

${\omega_{R}}^{3}\propto\frac{C_{g}+C_{m}\pm\sqrt{{C_{g}}^{2}+{C_{m}}^{2}}}{C_{m}C_{g}}$. (5)

Then, substituting the expressions of $C_{g}$ and $C_{m}$into equation (5), we can obtain

${\omega_{R}}^{3}\propto\frac{t}{c_{1}\varepsilon_{2}\varepsilon_{0}}\frac{1}{D^{2}}+\frac{P}{\varepsilon_{0}h}\frac{1}{D}-\frac{1}{\varepsilon_{0}h}\pm\sqrt{\left( \frac{t}{c_{1}\varepsilon_{2}\varepsilon_{0}}\frac{1}{D^{2}} \right)^{2}+\left( \frac{P}{\varepsilon_{0}h}\frac{1}{D}-\frac{1}{\varepsilon_{0}h} \right)^{2}}.$ (6)

Since $(1/D^{2}) \gg(1/D) \gg1$, the terms with small values in Eq. (6) can be ignored and we have

${\omega_{R}}^{3}\propto\frac{2t}{c_{1}\varepsilon_{2}\varepsilon_{0}}\frac{1}{D^{2}}\propto\frac{1}{D^{2}}.$ (7)

Considering $\lambda_{R} = 2\pi c/\omega_{R}$, we can obtain the relationship

$\lambda_{R}\propto D^{\frac{2}{3}}.$ (8)

Similarly, because $L_{m}$ and $C_{m}$ are both independent of the height h of the Cr nanodisks, it can be obtained from Eq. (3) that

${\omega_{R}}^{2}\propto\frac{1}{C_{g}}\frac{1}{L_{e}}.$ (9)

By substituting the expression of $L_{e}$ into Eq. (9), we have

$\frac{1}{{\lambda_{R}}^{3}}{{=\omega}_{R}}^{3}\propto\frac{1}{C_{g}}=\frac{P-D}{\varepsilon_{0}D}\frac{1}{h}\propto\frac{1}{h}.$ (10)

Then, we can obtain

$\lambda_{R}\propto h^{\frac{1}{3}}$. (11)

Consequently, the resonance wavelength of the MP resonance increases as *h* or *D* increases, which agrees with the results shown in Fig. 7(c) and 7(d).

Bibliography

1. H. Xu, L. Hu, Y. Lu, et al., “ Dual-Band Metamaterial Absorbers in the Visible and Near-Infrared Regions,” J. Phys. Chem. C, vol.123, pp. 10028-10033, 2019. https://doi.org/10.1021/acs.jpcc.9b00434
2. P. Ekkels, X. Rottenberg, R. Puers, et al., “Evaluation of platinum as a structural thin film material for RF-MEMS devices,” J. Micromech. Microeng., vol. 19, no. 065010, 2009. https://doi.org/ 10.1088/0960-1317/19/6/065010
3. Y. Matsuno and A. Sakurai, “Perfect infrared absorber and emitter based on a large-area metasurface,” Opt. Mater. Express vol. 7, pp. 618-626, 2017. https://doi.org/10.1364/OME.7.000618
